# Effects of transcranial direct current stimulation (tDCS) associated with balance training in individuals with Parkinson's: Study protocol for a randomized clinical trial

**DOI:** 10.1016/j.mex.2024.103014

**Published:** 2024-11-10

**Authors:** Raynara Fonseca dos Santos, Guilherme Peixoto Tinoco Areas, Fernando Zanela da Silva Areas, Pedro Porto Alegre Baptista, Ayrles Silva Gonçalves Barbosa Mendonça, Renato Campos Freire Junior

**Affiliations:** a Postgraduate Program in Human Movement Sciences, Federal University of Amazonas, Manaus, AM, Brazil; b Physiology Sciences Laboratory, Federal University of Amazonas, Manaus, AM, Brazil; c Baylor Scott and White Research Institute, Dallas, TX, USA; d Baylor Scott and White Institute for Rehabilitation, Dallas, TX, USA; e Laboratory of Neurorehabilitation and Neuromodulation, Federal University of Espirito Santo, Vitoria, ES, Brazil; f Laboratory of Assistive Technology and Movement Analysis (LABTAM), Federal University of Amazonas, Manaus, AM, Brazil

**Keywords:** Postural balance, Cerebellum, Transcranial direct current stimulation, Exercise therapy

## Abstract

The present study aimed to investigate the effects of balance training associated with cerebellar tDCS on postural control in individuals with PD. This is a randomized clinical trial in which individuals were allocated to an experimental group (EG) or placebo group (PG), in which a conventional protocol of 10 Physiotherapy sessions for locomotor training and postural control was applied. In the EG, tDCS was applied, with a current setting of 1.5 mA for 20 min simultaneously with postural control training. In the PG, tDCS was applied in sham mode, with the same electrode positioning and the same number of sessions as the EG. The sample compared 34 individuals with PD (EG: 17; PG: 17).Cerebellar tDCS associated with balance training may help improve postural control and balance in walkers with Parkinson's Disease. The hypothesis is, if walking improve, the benefits may be accompanied by better balance and reduced fear of falling, and individuals may experience greater free-living physical activity at home and in the community.

## Graphical abstract

**Figure:**
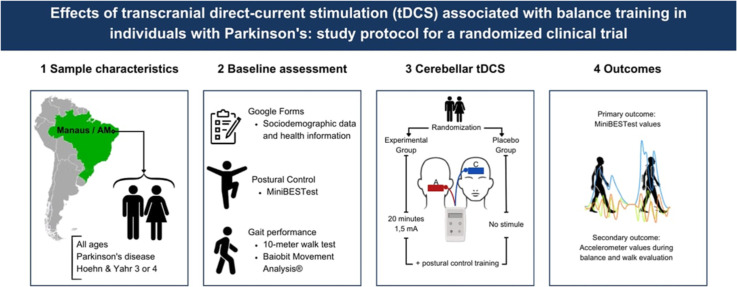

Specifications table

**Table:** 

Subject area:	Neuroscience
More specific subject area:	**TRANSCRANIAL DIRECT CURRENT STIMULATION (tDCS) ASSOCIATED WITH BALANCE TRAINING IN INDIVIDUALS WITH PARKINSON'S**.
Name of your protocol:	**EFFECTS OF TRANSCRANIAL DIRECT CURRENT STIMULATION (tDCS) ASSOCIATED WITH BALANCE TRAINING IN INDIVIDUALS WITH PARKINSON'S: STUDY PROTOCOL FOR A RANDOMIZED CLINICAL TRIAL**
Reagents/tools:	tDCS: Neuromodulator from Microestim Foco Research, NKL, Santa Catarina, Brazil. Baiobit Movement Analysis® (BTS Bioengineering S.P.A., Italy) All acceleration data will be recorded by sampling at 100 Hz frequency, transmitted to a notebook via Bluetooth and processed by the Rivelo software program (BTS Bioengineering Group, S.P.A., Italy
Experimental design:	A prospective, randomized controlled trial with concealed allocation, blinded assessors (participants and therapists), and intention-to-treat analysis will be carried-out (Figure 1). Community-dwelling people with PD will be recruited from the general community, by means of advertisements and by screening reference outpatient clinics. Participants of both sexes will be included, regardless of age, with a clinical diagnosis of PD, classified as 3 or 4 Hoehn & Yahr scale^18^, and who agreed to participate in the study with consent. Individuals with neurological disorders other than PD and with cognitive deficits that prevented them from carrying out the assessments were not included, and individuals with scores lower than 8 on the 10-CS were not included^19^
Trial registration:	Brazilian Registry of Clinical Trials (RBR-52y3zgd
Ethics:	The study was conducted in line with recommendations from the Declaration of Helsinki and received ethical approval from the Ethics Committee of the Federal University of the Amazonas, Brazil [CAAE 54,743,121.5.0000.5020].
Value of the Protocol:	Cerebellar tDCS associated with balance training may help improve postural control and balance in walkers with Parkinson's Disease. The hypothesis is, if walking improve, the benefits may be accompanied by better balance and reduced fear of falling, and individuals may experience greater free-living physical activity at home and in the community. The goal of protocol is to investigate the effects of balance training associated with cerebellar tDCS on postural control in individuals with PD.

## Background

The reduction or insufficiency of dopamine in Parkinson's disease (PD) triggers significant impacts on the extrapyramidal system, manifesting itself through symptoms such as tremors, plastic hypertonia, bradykinesia, freezing and decreased postural reflexes [1]. These manifestations tend to result in disturbances in balance, gait and postural control. As consequence, increases falling risk and a deterioration in the quality of life of affected individuals [2,3].

Several therapeutic approaches are used in the treatment of PD, with emphasis on pharmacological[4] and surgical interventions [5]. Furthermore, it is important to highlight that physical exercise is supported by a substantial level of evidence in the therapeutic management of individuals affected by PD [6]. This approach demonstrates the ability to improve several aspects affected by the disease, covering issues related to physical capacity, inactivity, gait, posture, transfers, balance and fall prevention [6,7]. Physical exercise has the potentially improves both motor aspects, such as gait, balance and strength, as well as non-motor aspects, such as depression, apathy, fatigue and constipation, associated with PD. Furthermore, it may contribute to the mitigation of secondary complications resulting from immobility, such as cardiovascular problems and osteoporosis [8,9].

Among the resources available for the treatment of impairments linked to PD is Transcranial Direct Current Stimulation (tDCS). In general terms, tDCS consists of the application of low-intensity direct electrical current (1–4 mA) through two surface electrodes: a cathode (negatively charged) and an anode (positively charged). This approach differs from Transcranial Magnetic Stimulation (TMS), which consists of applying short-duration magnetic pulses to induce electrical currents in the brain [[10], [11], [12], [13]]. tDCS produces a potential difference between the applied electrodes, thus generating changes in the neuronal membrane potential that is located within the resulting electric field. The effect produced depends on both the direction of the current (anodal or cathodal) and the axonal orientation, resulting in neuronal depolarization or hyperpolarization. In general, anodal tDCS tends to increase neuronal excitability, while cathodal tDCS tends to decrease it [11,12,14].

The cerebellum plays a relevant role in motor control [15]. It is related to the control of the vestibular system, proprioception, cognition and motor programming, receiving visual and auditory input and sending efferent fibers to the thalamus, red nucleus and cortex (motor, pre-motor and pre-frontal cortex) [16]. tDCS has been widely used in the motor cortex, however, Mello [17] suggests that applying tDCS in the cerebellar region could be an alternative to stimulate the efferent pathways of the cerebellum to act on the cortical structures involved in motor processing and, in this way, influence the motor learning process [17].

Therefore, the objective of the present study is to investigate the effects of a balance training program associated with cerebellar tDCS on postural control in individuals with PD.

The specific research questions are:

• In people with Parkinson's Disease, is balance training associated with tDCS superior to balance training alone for improving walking (balance, postural control), mobility and falls?
• Are any benefits maintained beyond the intervention period?

## Description of protocol

Methods details

## Ethical approval

The study was conducted in line with recommendations from the Declaration of Helsinki and received ethical approval from the IRB of the Federal University of the Amazonas, Brazil [CAAE 54,743,121.5.0000.5020].

## Sample size and subject recruitment

For the sample calculation, the primary outcome was the total MiniBESTest value. The value of the minimum clinically significant difference is 3.4 points in the total MiniBESTest value [18]. Using this value, for the statistical calculation of ANOVA two-way (between-within factors) an effect size of 0.25 Cohen's F was found. Therefore, we calculate using an effect size of 0.25 Cohen's F, type I error (alpha) of 5 %, type II error (beta) of 80 %, 2 groups, 3 moments (before training, after training and follow-up) and a correlation between repeated measurements of 0.5. After the calculation we arrived at the value of 14 patients per group. Accepting the possibility of 20 % possible sample loss, the selection of 17 patients per group was considered. For the calculation, the statistical software G*Power 3.1 (Universitat Kiel, Kiel, Germany) was used.

**Fig. 1:**
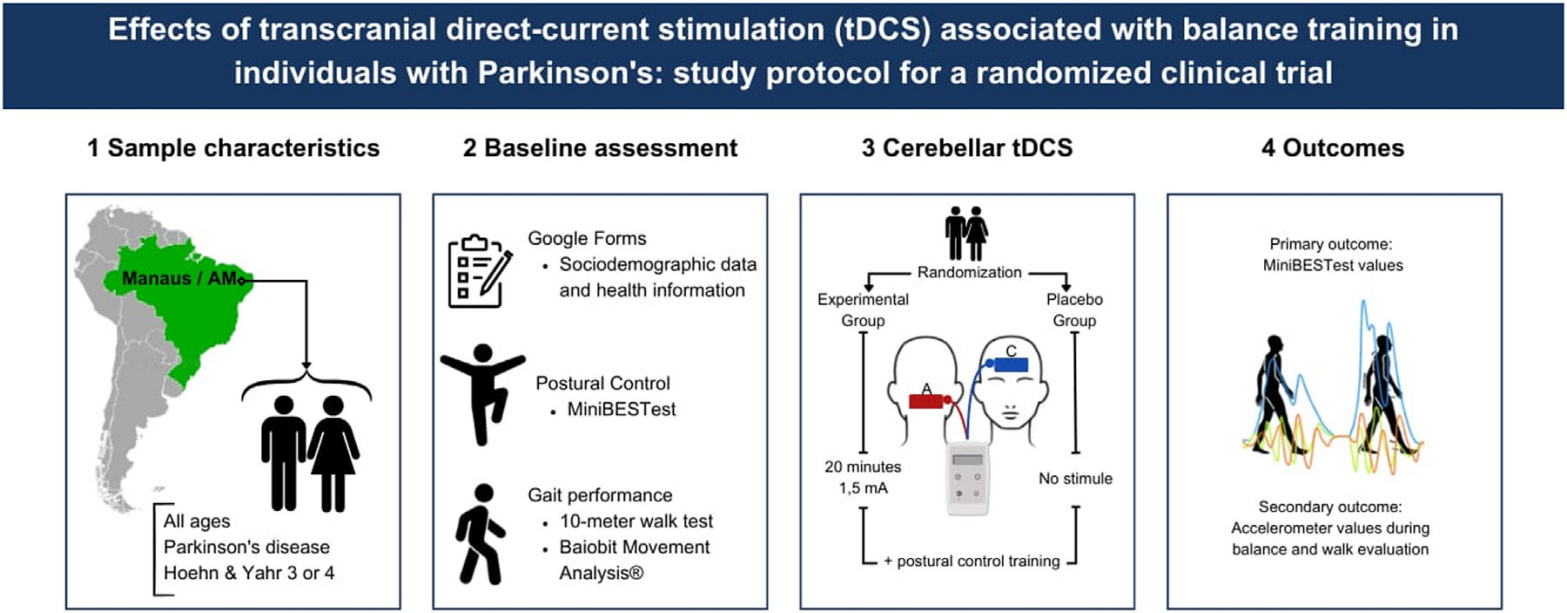
Illustrative figure of the methodological stages of the study.

Community-dwelling people with PD will be recruited from the general community, by means of advertisements and by screening reference outpatient clinics. Participants of both sexes will be included, regardless of age, with a clinical diagnosis of PD, classified as 3 or 4 Hoehn & Yahr scale [19], and who agreed to participate in the study with consent. Individuals with neurological disorders other than PD and with cognitive deficits that prevented them from carrying out the assessments were not included, and individuals with scores lower than 8 on the 10-CS were not included [20]. The study was written based on the CONSORT checklist [21] and included a total of 4 stages: Initial assessment (AV1), Intervention, Immediate Post-intervention Assessment (AV2) and Reassessment (follow-up) (Fig. 1). After providing a signed consent form, individuals were invited to participate in the assessment and intervention protocols described below.

## Subject data collection

Informed consent was obtained from every subject enrolled in the study.

Measurements and interventions will be conducted during the on phase of medication. Analyses of inclusion criteria, getting informed consent, data collection, and statistical analyses will be carried out by researchers, who will be blind to group allocation. All the participants will be evaluated and receive all the information regarding the interventions in a research laboratory. The study obtained ethical approval from the Institutional Research Ethical Committee (CAAE 54,743,121.5.0000.5020) of the *Universidade Federal do Amazonas*, Manaus, Brazil. The trial was prospectively registered at the Brazilian Registry of Clinical Trials (RBR-52y3zgd).

### Assessment

The initial assessment consisted of sociodemographic data and health information of the participants, being carried out via telephone contact using a Google Forms form as a form of screening, together with assessments of the severity of the disease using the Hoehn & Yahr, cognitive level through the 10-Point Cognitive Screener and the perception of physical activity level through the International physical activity questionnaire (IPAQ). The variables of interest for sociodemographic and health data will be: age, sex, education, income, fear and occurrence of falls, freezing and duration of illness.

Postural control performance will be assessed using MiniBESTest [18]. Individuals underwent the 10-meter walk test to assess gait performance [[22], [23]]. For each participant, 3 collections will carry out, and the arithmetic mean of the recorded variables will considered for analysis. The individual was instructed to walk at their usual speed.

The variables of interest were: speed (m/s), cadence (steps/min), stride length (meters), step length (meters), step duration (seconds), support phase (% of the cycle), swing phase (% of cycle), individual support. Gait performance assessment data will be recorded by Baiobit Movement Analysis® (BTS Bioengineering S.P.A., Italy), a portable wireless inertial system with wearable sensors, which weighs 37 g and measures 70 × 40 × 18 mm, composed of a triaxial accelerometer (16 bits/axes) with multiple levels of sensitivity (±2, ±4, ±8, ±16 g), a triaxial gyroscope (16 bits/axes) with multiple sensitivity levels (±250, ±500, ±1000, ±2000°/s) and a triaxial magnetometer (13 bits, ± 1200 µT). To assess gait, the device is fixed with a semi-elastic belt to the posterior region of the pelvis of the subject being examined, approximately at the level of the L5-S1 vertebrae.

All acceleration data will be recorded by sampling at 100 Hz frequency, transmitted to a notebook via Bluetooth and processed by the Rivelo software program (BTS Bioengineering Group, S.P.A., Italy) [24,25].

Baiobit will be also used to evaluate the final phase of MiniBESTest, which includes carrying out the normal and dual-task Timed up and go (TUG test) (TUG DT) (subtraction of 3 by 3), with 3 collections also being carried out. The variables of interest will be the duration of the test (time), duration of the phases: sitting to standing, turning back, turning to sitting and standing to sitting.

### Blinding and randomization

The randomization will be generated by computer by an independent researcher not involved in the recruitment process or in the application of the intervention protocol, through randomization in blocks of 4, in order to guarantee balance in terms of the number of participants between the groups, being: experimental (EG), who received the balance training protocol associated with the application of tDCS, or placebo (PG), who received the balance training protocol associated with tDCS-placebo.

## Primary outcome measurements

The device used for tDCS was the Microestim Foco Research (NKL, Santa Catarina, Brazil). The devices has 2 types of stimulation: Real and Sham, the latter being a simulation of electrical stimulation, but which does not completely provide the stimulus to the patient. This technique is also known as “placebo stimulation” and aims to make the patient believe that they are receiving real stimulation, although the energy provided by the equipment is significantly lower than the energy provided by a normal-type stimulus.

To access Normal or Sham type stimulation on the equipment, the operating Physiotherapist enters a valid Use Code made up of 5 numbers. Microestim has 200 use codes, 100 codes for use with the real stimulation and 100 codes for the sham stimulation. These codes being provided to Physiotherapists by the independent researcher, ensuring the blinding of everyone involved in the research. Participants were identified by codes, to guarantee confidentiality throughout the research process.

### Intervention

In both groups, a conventional Physiotherapy protocol will be applied for locomotor training and postural control, based on the study of exercises [6] for the trunk and lower limbs, using a metronome, during the “on” time of the medication, with the aim of training semi-static and dynamic balance, provided by 10 sessions, spread over 4 weeks (Week 1: 3 sessions; Week 2: 3 sessions; Week 3: 3 sessions; Week 4: 1 session).

To apply tDCS, the individual was positioned sitting on a chair and the electrodes were inserted into sponges with saline solution, fixed with an elastic band. The anode electrode was positioned in the cerebellar region (OZ)^26^, and the cathode in the frontal region (fp1, fp2) [26], according to the International System 10–20 for insertion of Electroencephalography (EEG) electrodes [27,28].

After parameterization, the device will be attached to the patient's waist in a transparent material bag to visualize proper functioning. In the EG, tDCS was applied, with a current setting of 1.5 mA for 20 min, added to an initial period of 10 s of current rise and a final period of 10 s of current descent, simultaneously with postural control Training.

In the PG, tDCS was applied in placebo mode, with the same electrode positioning and the same number of sessions as the EG, however, the device remained in sham mode.

### Statistical analysis

To demonstrate the data, the results were described as mean ± standard deviation, median and interquartile range and percentage values. To calculate the cost of the dual task, the formula was used: [(TUG DT performance – TUG performance)/ TUG performance * 100 %] [29].

Initially, the Shapiro-Wilk test will be performed to assess the normality of the data and the Levene test to assess homogeneity. When the data did not show homogeneity of variances, the Welch test correction will be applied in the main analyses.

To analyze the characteristics of the groups, the *X*^2^ test and unpaired t student's test will be used. To analyze the primary and secondary outcomes, ANOVA two-way (between-within factors) *post hoc* Tukey will be used. To analyze the effect size of the unpaired t student's test Cohen's D will be used and to analyze the size of ANOVA two-way Cohen's F group will be used, and will be used the G*Power 3.1 software (University of Dusseldorf, Dusseldorf, Germany). For Cohen's F (Cohen's D), up to 0.10 (0.20) will be accepted as a no effect size, 0.11 – 0.24 (0.22 – 0.49) as a small effect size, 0.25 – 0.35 (0.50 – 0.70) as a moderate effect and equal to or greater than 0.40 (0.80) as a strong effect size. The intervention effect will be calculated based on intention-to-treat analysis. Values *p* < 0.05 were accepted as significant. The software used will be Jamovi 2.3 software (Jamovi project, Sydney, Australia) and Graphpad Prism 9.0 software (Graphpad, California, USA).

## Protocol validation

Data analysis for the protocol is currently ongoing. An example of a Excel data file (pilot study) for the analysis can be found in the Supplemental section..

## Limitations

This trial has some limitations. The experimental and control interventions consist of balance exercises delivered three times per week over one month, and, therefore, depend on participants’ motivation, adherence and commitment. Strategies to encourage participants to comply with the protocol, such as contracts and phone calls are planned.

## CRediT authorship contribution statement

**Raynara Fonseca dos Santos:** Conceptualization, Methodology, Writing – original draft. **Guilherme Peixoto Tinoco Areas:** Writing – review & editing. **Fernando Zanela da Silva Areas:** Writing – review & editing. **Pedro Porto Alegre Baptista:** Writing – review & editing. **Ayrles Silva Gonçalves Barbosa Mendonça:** Writing – review & editing. **Renato Campos Freire Junior:** Supervision, Writing – review & editing.

## Declaration of competing interest

The authors declare that they have no known competing financial interests or personal relationships that could have appeared to influence the work reported in this paper.

## Data Availability

No data was used for the research described in the article.

## Appendix Supplementary materials

Image, application 1
